# Circadian Blood Pressure as an Indicator for Cardiovascular Complications: CBP as an Indicator for Cardiovascular Complications

**DOI:** 10.31661/gmj.v14i.3604

**Published:** 2025-01-18

**Authors:** Zainab Al-Rikabi, Nihal Mohanad Lutfi

**Affiliations:** ^1^ College of Medicine, Ashur University, Baghdad, Iraq; ^2^ Department of Physiology, College of Medicine, University of Baghdad, Baghdad, Iraq

**Keywords:** Circadian Rhythm, Blood Pressure Fluctuations, Cardiovascular Complications, Hypertension, Circadian Disruption

## Abstract

The circadian variation in blood pressure is an important factor influencing various changes in the body over a 24-hour cycle and it has recently been in the focus of research for its ability to act as a sign of cardiovascular health. The following paper examines the interconnection, between the rhythmic pattern that characterizes blood pressure and how this affects cardiovascular events. It integrates newly appearing data that explain the applicability, prognostic, and therapeutic importance of those changes in recognizing and preventing the cardiovascular comorbidities. In fact, research confirms the affiliation between the daily fluctuation of circulatory pressure and the enhanced propensity for cardiovascular disorder, underlining the need for round-the-clock blood pressure regulation. Furthermore, there is clear evidence connecting disruption of the standard diurnal cycle, for instance due to abnormally sleeping or shift work, with increased vulnerability of hypertension and unfavorable cardiovascular outcomes. It is also seen that such patterns of reasons are possible if there is an innovative therapy for addressing cardiac ailments.

## Introduction

The natural biological timing system known as the circadian rhythm regulates and coordinates several physical functions, including the blood pressure beat cycle throughout 24 hours [1]. The circadian rhythm refers to the intrinsic organic timer that controls the slumber-awakening rhythm and additional bodily functions in alive entities. [2][3]. This internal fluctuation that has received considerable interest in human health, especially within the sphere of cardiovascular disease (CVD) [4]. Hypertension is a pervasive and formidable risk factor for CVD with a plethora of evidence supporting its causal relationship with various CVD manifestations, including arrhythmia, coronary heart disease, congestive heart failure [5][6]. The age-adjusted frequency of high blood pressure in the American mature populace was approximated to be 115.3 million American grown-ups suffering from elevated blood pressure in 2017-2020 [7]. Blood pressure follows distinctive circadian rhythm, with a diminution throughout slumber and a precipitous escalation in proximity to the moment of arousal [8][9][10]. This pattern is influenced by the innate circadian rhythm, which is controlled by an internal clock that responds to light and darkness [9]. Driven by an endogenous circadian rhythm, which causes the behaviour of BP to fluctuate throughout the day and night, any change or variation to this particular set up could pose some sort of threat to the health of an individual and may lead to hypertension or any other cardiovascular diseases. The circadian rhythm of blood pressure is also affected by various factors, including age, lifestyle, and underlying medical conditions [8][9][10]. As the current literature on circadian blood pressure and its relationship with cardiovascular complications is limited by a reliance on correlational evidence, with a lack of detailed understanding of the causality between circadian rhythm and hypertension, we aimed to explore the interconnection between the rhythmic pattern of blood pressure. What makes this study novel is its focus on the effects of disrupted circadian rhythm indicators on the development of hypertension and its complications.

## Circadian Rhythm

Circadian blood pressure illustrates the variability that exists in the cardiovascular system and its changes for a regular 24-hour cycle. As with any other thing that can be tested on an individual, the blood pressure may also have fluctuations depending on the time of the day, in the same way that one’s energy level or mood may differ at a certain time of the day. It for instance, is relatively high at morning and reduces during the night whilst sleep. The diel oscillation is regulated by a complicated network comprising the hypothalamic nuclei in the cerebral cortex and is harmonized by extrinsic temporal cues, such as luminosity-obscurity patterns, nourishment, and communal surroundings [2][11]. The intrinsic period of the circadian clock varies from one species to another and from one person to another, with people possessing a natural cycle marginally exceeding 24 hours [12]. The circadian rhythm acts as an internal timer, coordinating the function of multiple biochemical and physiological systems, and its disruption can lead to various health problems [13]. The discovery of the genetic origin of the circadian clock in Drosophila melanogaster by Konopka and Benzer in 1971 marked a significant milestone in the field [14][15]. Since then, researchers have made groundbreaking discoveries using genetic screens in Drosophila, identifying core clock genes and their molecular functions [16].

The timing of melatonin secretion, particularly the faint serotonin commencement, is a firmly-founded signifier of diel cycle somnolence anomalies [17]. Other indicators of circadian rhythm include the phase angle of entrainment, free-running circadian period, mid-sleep on work-free days, and the score from the Morningness-Eveningness Questionnaire [17]. Furthermore, alterations in the circadian excretion of urinary variables, such as catecholamine metabolites and adrenal cortical hormones, can also serve as indicators of circadian rhythm [18].

## Pathophysiology of hypertension

The regulation of blood pressure is a complex process that involves the integrated actions of multiple cardiovascular, renal, neural, endocrine, and local tissue control systems [19][20][21]. The kidney plays a critical role in regulating blood pressure, and renal mechanisms are often implicated in the development of hypertension [19][20][21]. Additionally, the compassionate neural network is also implicated in the abnormal physiology of high blood pressure, especially in adiposity-related high blood pressure [20][21]. Kidney regulation of exterior fluid volume and kidney blood flow pressure is complexly connected to the modulation of sodium elimination which in turn affects the function of various blood vessel-constricting systems, including the renin-angiotensin-aldosterone mechanism (RAAS) [22]. RAAS is one of the pathways that was selected in our research for evaluation of its link with circadian rhythm as well as the autonomic nervous system. The autonomic nervous system is one of neural components regulating blood pressure by controlling vasomotor tone, heart rate, and cardiac output [23][24]. The brain, specifically the medulla oblongata, is responsible for maintaining life-sustaining, resting levels of blood pressure and adjusting it in response to changes in regional blood flow and environmental stimulation [23][24]. Circulatory performance is also a vital element that influences arterial tension immediately, and it is a primary tactic for medically managing arterial tension to modulate contraction/dilation function of arterial conduits, including frictional force-induced liberation of nitrogen monoxide (NO) from the intima and the inherent vascular reaction [25].

## Circadian rhythm, RAAS, and hypertension

The RAAS modulates cardiovascular tension, liquid equilibrium, and ionic stability [26][27]. Recent studies have highlighted the importance of the circadian rhythm in modulating the activity of the RAAS [28][29]. The RAAS exhibits a natural circadian variation, with peak activity typically occurring in the early morning hours and decreasing at night [26]. Additionally, the cerebral angiotensin-converting enzyme network (ACE) might influence melatonin production, which possesses firmly-founded functions in controlling cycles [30]. Circadian rhythm disruption has been shown to have a profound impact on the RAS, leading to alterations in angiotensin II levels, which play a crucial role in regulating blood pressure and cardiovascular function [31]. Furthermore, research has demonstrated that angiotensin II infusion can disrupt the circadian rhythm of mean arterial pressure in both male and female rats [31].

## Circadian rhythm, autonomic nervous system, and hypertension

An investigation has revealed that autonomic neural system malfunction is linked to an intensified diel hemodynamic pressure differential and more fluctuating hemodynamic pressure over a 24-hour period in individuals with primary arterial hypertension [32]. The daily cycle of hemodynamic pressure is controlled by the suprachiasmatic nucleus, which adjusts the involuntary neural system activity targeted at the cardiac organ and vascular tubes 4. Interruption of physiological cardiovascular daily cycles has significant medical consequences, as it is correlated with increased sickness and death rates [33]. Moreover, an examination has shown that the central sympathoexcitatory pathway to the upper thoracic spinal cord plays a vital role in maintaining normal daily hemodynamic pressure rhythm in humans [34]. Also, the connection between nighttime hemodynamic pressure and organ damage has been discovered to be curvilinear, emphasizing the importance of considering daily cycles in the treatment of arterial hypertension [35].

## Circadian rhythm, Vascular function, and hypertension

The endothelial function, which is responsible for regulating vascular tone, is also influenced by the circadian rhythm, with impaired endothelial function being a hallmark of hypertension [36][37]. The endothelial function is closely linked to the circadian rhythm, with the production of nitric oxide and other redox species playing a crucial role in regulating vascular contractility [38]. The peripheral circadian clock mechanism has been shown to control the enzymes involved in generating these species, and disruption to the clock can result in endothelial and vascular dysfunction [38].

## Circadian rhythm genetics and hypertension

The circadian rhythm oscillation is controlled by a collection of essential "timekeeper genes" that constitute a reciprocal loop of genetic transcription and interpretation [39][40]. These genetic sequences, including hClock and hPer2, have been linked to human somnolence disturbances and have been demonstrated to be articulated in a periodic manner in peripheral blood mononuclear cells throughout the somnolence/wakefulness and diel cycles [39][41]. The articulation of these genetic sequences is influenced by the suprachiasmatic nucleus (SCN), which is the primary diel pacemaker of the cerebral cortex [41]. However, recent evidence proposes that functional timekeepers exist outside the SCN, and that the diel clock influences the patterns of genetic expression and cellular operations in various tissues [42][43]. Investigations have shown that BMAL1, a core timekeeper protein, might be involved in regulating diel blood pressure and is implicated in various sclerotic disorders [44]. A high-sodium diet has been found to suppress the articulation of BMAL1, leading to diel changes in hypertension and increased susceptibility to atrial fibrillation [45]. Furthermore, research has demonstrated that BMAL1 is associated with susceptibility to gestational diabetes mellitus and is involved in the regulation of solute handling and blood pressure control in the renal organs [46][47]. Investigations have shown that the insertion/deletion (I/D) polymorphism of the ACE gene can influence ambulatory blood pressure levels and diel blood pressure rhythm [48][49]. Specifically, studies have found that individuals with the DD genotype tend to have impaired diel blood pressure variation, characterized by a lack of nocturnal blood pressure dip, which is associated with increased mortality rates in subjects with diabetes [50].

## Clinical Evidence

Evidence have shown that this circadian rhythm is present in both normotensive and hypertensive individuals, with the peak-to-trough amplitudes ranging from 3 to 6 mm Hg for systolic blood pressure and 2 to 3 mm Hg for diastolic blood pressure [51].

A cross-sectional study found that circadian variation in blood pressure was associated with pathologic cardiovascular parameters, and significant differences were observed between genders [52]. Another study on chronic kidney disease patients revealed that circadian blood pressure variability was linked to various factors, including daytime and nighttime blood pressure [53]. Furthermore, García-Ortiz et al. study has shown that Habitual bodily exercise, assessed precisely and by personal account, can affect the daily cycle of 24-hour mobile arterial pressure [54]. Also, the connection between daily cycle of bloodstream pressure and vascular impairment in necessary high blood pressure has been examined in patients with necessary high blood pressure, with findings indicating that daily cycle is linked to brachial-ankle pulse wave speed and upper-arm artery flow-mediated expansion [55].

The daily cycle of bloodstream pressure is altered from a reducer to a non-reducer pattern in rotating workers with high blood pressure, and this alteration can occur swiftly, within the first day of nighttime work [56][57]. Furthermore, social time-zone displacement evaluation may be a valuable measure in rotating work populations, where the extent of daily cycle mismatch may be greater than in the overall population [58]. The impact of blood pressure-lowering medications on daily cycles of bloodstream pressure and cardiac rhythm has also been researched, with findings suggesting that calcium pathway blockers, heart-rate regulators, and ACE suppressors can reduce bloodstream pressure throughout the day without altering the daily bloodstream pressure cycle [59][60].

The daily cycle of cortisol has been demonstrated to impact arterial tension in robust individuals, with reduced daytime fluctuation in corticosteroid correlated with reduced daytime fluctuation in blood pressure [9]. Furthermore, Matsumura et al. has demonstrated that the administration of exogenous cortisol can modulate the circadian variation of blood pressure in patients with hypopituitarism [61]. Additionally, studies have found that blunted circadian cortisol rhythms are associated with poor cardiovascular health even in children and may reflect circadian misalignment [62]. Azmi and others discuss the aspects of cortisol, a hormone that has daily fluctuations, and stresses that it is most elevated in the morning and interacts with cardiovascular processes [63].

A study has shown that melatonin plays a crucial role in regulating blood pressure, with studies demonstrating that repeated melatonin intake can reduce nocturnal blood pressure in patients with essential hypertension [9]. Additionally, there is a link between melatonin and blood pressure regulation as impaired nocturnal melatonin secretion has been observed in non-dipper hypertensive patient [64]. The circadian rhythm of melatonin has also been shown to impact blood pressure, with disturbances in this rhythm potentially contributing to the development of hypertension [64][65][66]. Furthermore, melatonin supplementation has been found to improve sleep quality and reduce blood pressure in patients with essential hypertension [65][66].

## Circadian rhythm of blood pressure and cardiac complications

The relationship between circadian blood pressure and cardiac complications has been extensively studied, with research indicating that there is a significant correlation between the two. The morning surge in blood pressure has been identified as a potential trigger for myocardial infarction, with the risk of cardiovascular events increasing during this time [67][68]. Furthermore, research has suggested that the circadian rhythm of blood pressure may play a role in the pathophysiology of myocardial infarction, with factors such as acute variations in blood pressure, heart rate, and platelet aggregability contributing to the increased risk of plaque rupture and intracoronary thrombosis [67]. The alteration in diurnal blood pressure patterns has been identified as a significant risk factor for congestive heart failure [68]; while cognitive impairment in heart failure patients has been associated with abnormal circadian blood pressure rhythms [69]. Research has shown that there is a significant correlation between the circadian rhythm of blood pressure and the incidence of aneurysm rupture [70]. Specifically, it has been observed that the risk of aneurysm rupture is higher during periods of low atmospheric pressure [70]. Furthermore, Seguchi et al. have also demonstrated that there is a circadian variation in the onset of acute aortic dissection, with a higher incidence of events occurring during the early morning hours [71]. The circadian rhythm of blood pressure, which typically peaks in the early morning and decreases at night, may play a crucial role in the development and rupture of aneurysms [71].

## Relationship between CVDs and circadian blood pressure

The relationship between CVDs and circadian blood pressure involves several significant concepts:

### Circadian Rhythm Disruption

Vividly, circadian rhythm disturbances including interrupted sleep, shift work, or irregular sleeping patterns especially at night have been associated with hypertension which may hence cause cardiovascular ill-health [72].

### Impact on Heart Health

Everyday rhythms in blood pressure, in particular their non-stable pattern, have been reported to be risk factors for adverse outcomes such as heart attacks, stroke, and other cardiac events [73].

### Non-Dipping and Reverse Dipping

High BP at night or marked increase during sleep or no decrease in BP during sleep is another indicator associated with higher cardiovascular risks [74].

### Cardiorenal Risks

It also well-established this disrupted circadian blood pressure rhythm is an independent and powerful predictor of adverse outcomes in both the heart and kidneys. This relationship proves the close relationship between self-measured blood pressure at different times of the day and cardiovascular/renal disease related diseases [75].

### Clinical Implications of Abnormal Patterns of Circadian Blood Pressure Fluctuations

Further, Giles (2006) confirms that early morning blood pressure rise increases the risk of cardiovascular events through increasing rate pressure products especially for those in the elderly age bracket. This highlights the utility of normal oscillation of morning BPs to be appreciated and controlled.Research by Sun et al. aimed at essential hypertension reveals that the ABCC and AVSP are associated with disturbed circadian rhythms of blood pressure about vascular function [76].

The study by Sun et al. showed that decreasing nocturnal SBP can be used predicting baPWV/FMD of circadian hypertension [55]. Zhang et al. (2014) note that most individuals especially those in developed world societies have disrupted daily rhythms due to such factors as shift work, and evening exposure to artificial light among others, which increases the risk of cardiovascular diseases and the metabolic syndrome. This work underscores the utility of circadian biology in clinical applications and opens new frontiers for therapeutic prospect [73]. The study by Sun et al. (2023) found that missing this internal clock of blood pressure can lead to more frequent and serious cardiovascular events such as heart attacks, strokes or hypertension complications. Their investigation supports prior research attributing circadian irregularity and instability of blood pressure on enhanced cardiovascular risks; therefore, the clinical value of monitoring blood pressure fluctuations throughout the day is well validated in the literature [55][73].

### Pattern Irregularities and Risks

As it can clearly noticed between the non-dipping and reverse dipping: As it can be noted while comparing the non-dipping and reverse dipping patterns as per the following research of Sun et. By 2023, such situations/states make the patient with cardiovascular risk: vulnerable Any further and future activities and developments will further improve the situations/states which will also And any further and future activities or developments will further improve the situations/states which will similarly Such irregular ups and downs caused anxiety on a sinister variable of cardiovascular incident that would need concern at a lower capacity for general cardiovascular fitness in case the sleep wake cycle is distorted [55][73].

Disruption in asynchrony of blood pressure, and the influence of shift work further stress and destabilize circadian rhythms and thereby promote worsening of cardiovascular renal risk and is thought to be involved in the progression of acute kidney injury. Such relations suggest that comparing the circadian FBPs is vital in assessing the given risks about cardiovascular and renal health, as mentioned previously. The topic regarding the circadian blood pressure rhythms has been studied for a long time because the data obtained from such studies may significantly enhance the possibility of early prediction and diagnosis of cardiovascular diseases along with initiating necessary treatment in time [75][76]. Disruption in asynchrony of blood pressure, and the influence of shift work further stress and destabilize circadian rhythms and thereby promote worsening of cardiovascular renal risk and is thought to be involved in the progression of acute kidney injury. Such relations suggest that comparing the circadian FBPs is vital in assessing the given risks about cardiovascular and renal health, as mentioned previously. The topic regarding the circadian blood pressure rhythms has been studied for a long time because the data obtained from such studies may significantly enhance the possibility of early prediction and diagnosis of cardiovascular diseases along with initiating necessary treatment in time [75][76]. Furthermore, Clock-dick and other circadian research ‘clock’ readers provide understanding into circadian regulation of cardiovascular diseases with potential to develop interventions to treat abnormal DBP rhythmicity and its consequent cardio-vascular risks, as demonstrated by Zhang et al., 2014.

## Conclusion

These insights of patterns found in circadian blood pressure as indicators of cardiovascular complications add information to the understanding of comprehensive and predictive estimations of cardiovascular risks. By presenting some recent evidence concerning the relationship between circadian disruption and poor cardiovascular prognosis, our work underscores the importance of recognising the circadian nature of many clinical measurements. Thus, non-dipping and reverse-dipping patterns of circadian blood pressure were recognized as potential benchmarks for an increased risk of cardiovascular disorders. Knowledge of this relationship also assists in early risk identification and possible intervention pathways on how to prevent the risk factors that could lead to a higher cardiovascular risk More so, it opens up prospects for developing specific therapeutic approaches in an effort to deal with circadian-related blood pressure disorders.

To build on the findings of this review, it will be important to conduct additional study to establish how systemic clocks function to control blood pressure and their roles in cardiovascular-renal disorders. These findings will add to the knowledge of circadian biology and its influences on cardiac health, and will help to adapt or integrate circadian blood pressure tracking into everyday clinical practice. Combining these assessments may lead to enhanced classifications of cardiovascular risk and more individualized approaches to preventive cardiology in the future, which could ultimately help in creating new preventive measures and novel strategies for the effective management of cardiovascular diseases.

## Conflict of Interest

None.
